# Barriers and facilitators to primary care research: views of GP trainees and trainers

**DOI:** 10.3399/BJGPO.2021.0099

**Published:** 2022-04-20

**Authors:** Sarah Stephenson, Eugene Yee Hing Tang, Eugene Tang, Penny Williams, Hilary Allan, John Rouse, Morag Burton, Caroline Wroe, Richard Bellamy, Hannah Hesselgreaves

**Affiliations:** 1 Department of Leadership and Human Resource Management, Faculty of Business and Law, Northumbria University, Newcastle upon Tyne, UK; 2 Population Health Sciences Institute, Newcastle University, Newcastle upon Tyne, UK; 3 North East and North Cumbria Clinical Research Network, Newcastle upon Tyne, UK; 4 Health Education North East, Newcastle upon Tyne, UK

**Keywords:** Continuing professional development, Postgraduate education, Cross-sectional survey, Primary care, Medical schools, General practice

## Abstract

**Background:**

Primary care plays an important role in the conception and delivery of transformational research but GP engagement is lacking, prompting calls for the promotion of academic opportunities in primary care.

**Aim:**

To identify potential barriers and facilitators among GP trainees and trainers in primary care research to inform support given by Local Clinical Research Networks (LCRNs).

**Design & setting:**

A cross-sectional online survey was developed and distributed by the CRN to GP trainees and trainers in the North East and North West.

**Method:**

The survey covered areas including demographics, career intentions, current and potential engagement with research, as well as their general understanding of research in primary care, which included barriers and facilitators to primary care research.

**Results:**

Trainees had low intentionality to pursue research and half of trainees did not engage with any research activity. Despite one in five trainees reporting intentions to include research in their career, only 1% would undertake a solely academic career. Medical school region was the only strongly associated factor with academic career intention. Just under 30% of trainers reported engagement in research, but far fewer (8.6%) were interested in contributing to research, and only 10% felt prepared to mentor in research.

**Conclusion:**

Among trainees, there is limited engagement in and intentionality to pursue research, and this was crucially reflected by responses from trainers. This study identified the need for LCRNs to assist with training in research mentoring and skills, funding opportunities, and to develop resources to promote research in primary care.

## How this fits in

In March 2021, the National Institute for Health Research Clinical Research Network (NIHR) CRN approved a National Primary Care Strategy designed to develop, promote, and facilitate high quality research in the primary care setting, which is integral to delivering health and care for the population’s benefit. A fundamental element of this strategy was workforce development. The initial work streams of the strategy included evaluation of the support required and pilots to provide learning and research opportunities for staff in the primary care sector (Theme D: Strategic Development of the Primary Care Workforce). The findings from this survey highlighted the challenges in workforce engagement and establishes a baseline for intervention.

## Introduction

General practice provides more than 300 million patient consultations each year.^1^ Primary care can play a significant role in the delivery of transformational clinical research, most recently evidenced by the PRINCIPLE public health study, a platform trial designed to evaluate treatments to improve recovery and reduce hospitalisation from COVID-19.

The UK is a leader for primary care research, in volume and citation rates of articles produced, when compared to international colleagues^2^ as research in general practice is an established discipline. However, prior to COVID-19, the number of GPs in England engaged in clinical research was in decline. The NIHR CRN consists of 15 LCRNs that coordinate and support the delivery of research, providing local resources and training. The Cumbria and North East Primary Care Strategy recommended building capacity and capability for research through exposing medical students and GP trainees to primary care research and LCRN delivery to develop future research leaders.^3^ In order to facilitate academic primary care research development and delivery, it is important to understand the intentions and requirements of both future GPs and the trainers who assist in their development and training. The CRN North East and North Cumbria (NENC) is in the top 5 LCRNs in terms of percentage of GP practices recruiting to clinical trials, but within the region there is variation in primary care research activity. This variation, and declining patient recruitment figures, is compounded by a lack of academic opportunities for GPs, a rapidly changing landscape of primary care provision, increasing workload, transfer of work from secondary care, and inadequate research funding. As a result, the NIHR CRN has recently launched a new primary care research strategy.^4^ The Royal College of General Practitioners has called for increased academic activity by developing research capability in general practice, including the pursuit of research *about* general practice on consultation patterns or the approach to management and treatment of complex needs.^5^

Medical Schools Council and Health Education England (HEE) recommended an increase in the availability and promotion of academic opportunities for medical students and GP trainees,^6^ however, medical students describe general practice as neither academically challenging nor a prestigious specialty;^7^ have limited awareness of academic primary care;^8^ and perceive the lack of mentorship as a barrier. This is also reported by family practice students in the US.^9^

The objective of this survey was to describe the academic career intentions of GP trainees and to identify the potential barriers and facilitators to primary care research among GP trainees and trainers, thus assisting the LCRN in deciding where to focus resources and training to facilitate the development of future academic GPs, and the offer of NIHR research as an option of care within communities.

## Method

### Participants and recruitment

A cross-sectional online survey was developed by NIHR CRN NENC in collaboration with HEE North and Northumbria University.

Potential participants were identified within the regions of the North East and North Cumbria, and the North West who were either undertaking specialty training in primary care or were a GP trainer. Invitations to participate were emailed to GP trainees and trainers via the Training Programme Directors in each region of HEE North. This included a brief summary of the project with an electronic link to the questionnaire. Questionnaires were sent to approximately 500 GP trainees and 320 trainers during February–March 2020. An implied consent model was used, which inferred that survey completion signals willingness to participate.

### Survey

Two versions of the questionnaire (42 item trainee and 35 item trainer survey, Appendix 1 and 2) covering comparable questions were sent via email to GP trainees and trainers, via HEE North. Due to the lack of validated questionnaires and/or measures in this area, the questionnaires were developed by the authors, using a combination of questions adapted from published literature exploring the issues.^10–13^ Both versions were piloted with a small sample of each group to establish face validity and to test the timing of questionnaire completion, resulting in minor amendments, which included an adjustment to some stem questions, and more detail about the number of sessions for clinical service. The final items covered: demographics, current engagement in research, career intentions, understanding and awareness of GP research and research training, perceptions of barriers and facilitators to research in primary care, and awareness and perceptions of NIHR CRN. Revised questionnaires were circulated as a web link (SmartSurvey) and were accessible for 3 weeks, with one reminder.

### Analysis

Data were collected in Excel and analysed in Excel and IBM SPSS Statistics (version 22) in May 2020. A summary analysis was performed to estimate and cross tabulate responses by trainees and trainers. Correlations were excluded because of the nominal nature of many of the data. χ^2^ tests of independence and estimated Cramer’s V examined associations between key items. This study reported significant relationships where associations related to career intentions were tested, and where the minimum count did not violate assumptions for the test. All other data presented are descriptive.

There were too few text comments to warrant the use of software to manage these data, so summaries on reasons for career intentions, and responders’ understanding of NIHR CRN activities were drawn from the questions that produced text responses. The approach to the analysis of the qualitative themes was to identify areas that may help interpret or contribute richness to the survey data and acknowledge that these data may not be representative.

## Results

A total of 167 GP trainees and 140 GP trainers completed the survey; this study presented descriptive results in these two groupings. Some participants did not complete all questions, so the number for all data presented is reported.

### Descriptive characteristics

Full descriptive characteristics are presented in Table 1. The GP trainees’ (*n* = 167) ages ranged from 25 to 54; they were split evenly across training years 1–3, and 13 had previously applied for an academic post. Many reasons for not moving directly from foundation to speciality training were given (including travel, work abroad, maternity, locum work, further study, other speciality training, trust grade work); of the 19 who did not completely foundation training, most trained abroad.

**Table 1.** Characteristics of trainee participants

Characteristic	*n*	%
**Age, years**		
Mean 32.6 [range 25–54, SD 5.7]	167	100

BSc	20	12.0
PGCert	10	6.0
PGDip	8	4.8
MSc	22	13.2
PhD	2	1.2
Member of a medical college	6	3.6
**Year of training**		
ST1	57	34.3
ST2	58	34.9
ST3	48	28.9
Other	3	1.8
**Applied for an academic post previously**		
Yes	13	7.8
No	153	92.2
**Medical school region** ^a^		
North East and North Cumbria	25	15.1
North West	34	20.5
Other^b^	44	26.5
Overseas	63	37.7

^a^Missing data n = 1. ^b^Includes London, East Midlands, East of England, West Midlands, Wessex, Yorkshire and Humber, Scotland and Wales.

### Research career intentionality

Trainees generally expressed low intentionality for pursuing a research post either in conjunction with clinical service or on its own, with 44% (*n* = 73) of participants stating that they would choose a career in clinical service with some teaching (Q20). Nonetheless, a significant minority (20%) have career intentions which include some research. Less than 1% stated that they would undertake a solely academic career, further confirming that a clear majority are interested in predominantly clinical service (Table 2). When asked to describe their reasons for choosing careers without an academic component it was evident that, while there was an interest in research, trainees’ enjoyment and perception of value lay with clinical and teaching work. Supplementary Box S1 offers such explanations as described by participants.

**Table 2.** Trainee career choice intention within general practice (*n* = 166)

Career choice	*n*	Response rate, %
Clinical service post only	11	6.6
Clinical service post with some teaching	73	44.0
Clinical service post with some research	9	5.4
Clinical service post with some teaching and research	26	15.7
Solely clinical academic research post	1	0.6
Change speciality	9	5.4
Undecided	33	19.9
May leave medicine	4	2.4

Thirty-eight per cent of trainees reported that they intended to undertake some form of academic career. Associations were explored to see whether this was influenced by whether the trainees had intercalated, what year of training they were currently in, and where they had trained (Table 3). There was a significant association between medical school region trained and intention to undertake an academic career (research or education), X^2^ = 35.8 (3, *n* = 166 [*n* = 1 respondent did not provide medical school region]), *P* = 0.02, with overseas trainees more likely to favour an academic career.

**Table 3.** Trainee intention to undertake form of academic career (research or education) presented with associations with intercalation, year of training, and medical school region

Survey item	Response category	*n*/total	%	Trainee intention academic career (univariate analysis)
(Q7) Intercalated?	Yes	23/164	14	Not significant
	No	141/164	86	
(Q13) Year of training	ST1	57/166	34	Not significant
	ST2	58/166	34.9	
	ST3	48/166	28.9	
	Other	3/166	1.8	
(Q11) Medical school region^a^	North East and Cumbria	10/25	40	x^2^=35.8(3, *n*=166), *P*=0.02
	North West	12/34	35.3	
	Rest UK	10/44	22.7	
	Overseas	31/63	49.2	

Because of small sample size and to avoid violation of minimum cell size Χ^2^ test of associations, the five variables in Q25 were merged to two: intention to undertake an academic career; and intention to undertake an academic career.

^a^Significant.

#### Research engagement

Engagement in research activity was also low, with nearly half of the trainees not engaging in any research, and only 18% either interested in contributing to, or conducting, research (Q16/Q18), as shown in Table 4. This lack of interest had implications for their recruitment of patients to research (1.6% trainees reported that they recruited patients). Trainers’ perceptions and awareness of their senior colleagues’ involvement in academic work largely mirrors their own involvement (Q13), with education and training (teaching) being a significant element compared to research, and one in five being perceived as not engaged at all. Nearly 30% of trainers reported that they do engage in research in some form, but less than 10% of trainers are actually interested in any part of the research process.

**Table 4.** Trainee and trainer research engagement

	Trainee self-reports of engaging in…		Trainee awareness of trainers’ engagement in...		Trainer self-reports of engaging in…		Trainer self-report of interest in…	
	*Number of responses*	*%*	*Number of responses*	*%*	*Numbers of responses*	*%*	*Number of responses*	*%*
Education and training	67	35.6	117	52.7	136	51.9	122	87.8
Recruitment of patients to research	3	1.6	16	7.2	41	15.6	6	4.3
Contributing to research	17	9.0	31	14.0	31	11.8	2	1.4
Designing and carrying out research	8	4.3	13	5.9	6	2.3	4	2.9
No research	93	49.5	45	20.3	48	18.3	5	3.6
**Total**	**188**	**100**	**222**	**100**	**262**	**100**	**139**	**100**

Participants could tick more than one response.

#### Factors influencing the pursuit of research careers

This study explored possible factors in training that may improve understanding of these low levels of interest, engagement, and career intentionality.

For GP trainees, funded time for research was the most important factor influencing their exploration of research opportunities (Q40); for trainers, role modelling was most important (Q33) (Table 5). Only 10% of trainers felt prepared to be a research mentor, with 63.5% stating they are unprepared or very unprepared. Although role modelling is key to encouraging academic careers, few felt equipped to mentor trainees in research.

**Table 5.** Factors influencing research careers

		Funded researchcourses	Funded research qualifications	Funded time for research	Role modelling	Mentor scheme	Attendconferences	More informationduring training
Trainee	*Number of responses*(%)	94(15.8)	93(15.6)	106(17.8)	81(13.6)	85(14.3)	53(8.9)	83(13.9)
Trainer	*Number of responses*(%)	41(10.8)	65(17.1)	52(13.6)	94(24.7)	49(12.9)	53(13.9)	29(7.1)

Participants could tick more than one response. Role modelling received the most votes for trainers (25%). Funded time for research received the most votes for trainees (18%).

The authors asked trainers whether they had heard of the NIHR CRN (Q31), and 35% were not aware of the CRN (6.4% were unsure). When trainers were asked about their understanding of the activities of the CRN, responses varied in depth and breadth of understanding among those who were able to articulate the CRNs role (Supplementary Box S1).

#### Research understanding among interested trainees

Finally, of those trainees who highly ranked an interest in research (Q17), most had a poor understanding of what research in primary care entails or a poor awareness of the opportunities to take part in research (Q34) (see Table 6). Those interested in the recruitment of patients were not aware of what research entailed or of the opportunities (although only two of the five wanted to know about opportunities). Of those interested in contributing to research, half reported they were aware of what research entails, and two-thirds would like to hear more about research opportunities. Of those interested in carrying out research, only one in four were aware of what primary care research entailed, and most wanted to hear more about opportunities.

**Table 6.** Trainee interest and understanding of research (*n* = 164)

Interest in research (the number of times the item was ranked first) (Q17):	*n* (%)	Understanding of research (Q34)	*n* (%)
Recruitment of patients	5 (3)	Aware of what research in primary care entails	0
		Aware of opportunities to take part in research	0
		Would like to hear about research opportunities	2 (1)
Contributing to research	18 (11)	Aware of what research in primary care entails	6 (4)
		Aware of opportunities to take part in research	3 (2)
		Would like to hear about research opportunities	12 (7)
Carrying out research	12 (7)	Aware of what research in primary care entails	3 (2)
		Aware of opportunities to take part in research	2 (1)
		Would like to hear about research opportunities	11 (7)

This highlighted that even though some show an interest, very few understand what research entails. This suggests that, while improved communication of research opportunities is desired, converting this into viable research activity will require significant awareness raising and education.

## Discussion

### Summary

The purpose of this service evaluation was to describe the academic career intentions of GP trainees, identify barriers and facilitators to engagement in primary care research, and assess ways in which the NIHR CRN can support trainers and trainees to engage. The survey had a 37% response rate: although participation in this research may be an indicator of participants’ general research interest, this response rate is higher than the rates of interest and engagement in research that this article reported. Few participants have intentions to pursue research as part of their career choice, particularly when compared to teaching. This was reflected by the low levels of engagement with research activity, and potentially explained by a limited understanding of what primary care research entails and the availability of individuals prepared to be role models in research. Nonetheless, a significant minority (20%) have career intentions that include some research, highlighting those participants as a potential group on which further research engagement efforts could be focused. Two observations are of note: the low engagement in research among trainers (and trainees’ awareness of their trainers’ lack of engagement), possibly contributing to trainees’ access to further understanding about the research process; and low levels of interest among trainers in research delivery, possibly contributing to trainees’ limited appreciation of taking part in research in primary care. Taken together, these findings about low levels of trainer engagement and interest may signal something about the value of research to trainees. The support and information offered by the CRN is also poorly understood or accessed. Limited understanding of what research entails, and trainers not acting as role models, were therefore identified as barriers to trainees’ pursuit of research careers. Funding (of time and qualifications) and being more informed about what research entails during their training, were evident facilitators.

### Strengths and limitations

This study gathered views from GP trainees across all years of training in two regions, as well as capturing the views of GP trainers; this provides a useful comparison to help characterise a part of the training environment relating to research engagement. The evaluation was regional, so it is unclear whether the participant experiences are nationally representative or whether the levels of CRN or institutional support for primary care research vary geographically. The sample contained a proportionately high number of overseas medical graduates, and their experiences of academia may differ. If they had more or less exposure to research during their medical training, this could impact how they viewed research. However, some of the principles obtained from this survey could assist other LCRNs when informing their own evaluations for their population, and the methodology and insight gained from this pilot study will help to inform a planned national survey of GP trainees and trainers in the future.

### Comparisons with existing literature

Early training experiences can have a significant influence on GP career intentions so a strategic approach to comprehensive careers (including non-clinical elements) has been called for.^14^ This study revealed how role modelling should be central to both academic career crafting and existing research and organisational cultures in GP practices, which may currently be discouraging professionals in primary care from research careers.^15^

Symonds *et al* highlighted both the challenges of role model availability, and the benefits of embedding non-GP research expertise into GP practices to build research capacity.^16^ The Healthcare Improvement Studies Institute ^17^ have encouraged an extension to the academic fellowship model by advocating the appointment of expanding academic expertise in GP practices. This supported this study's findings to look to the mentorship and training to generate a pro-research culture in primary care as a priority, alongside investment in promoting academic careers.

Some trainees have cited a lack of academic role models as a barrier to choosing general practice as a speciality.^18^ This study's findings further demonstrated that low levels of role modelling may exacerbate the recruitment challenges facing primary care, by discouraging potentially research-active trainees. Conversely, those specialities that are highly prized by trainees (for example, surgery) contain strong, inspirational role models. If increases in research capacity in general practice are to be realised, a focus on increasing role modelling capacity is needed,^19^ and educators should adapt their remit beyond the doctor as teacher role.

### Implications for research

Clearly, those who express an interest in research should be encouraged and supported. Investment in education to improve research understanding and skills should be a priority. In addition to embarking on initiatives required to develop a new generation of research-active GPs from the existing trainee population, it might be prudent to consider how research-active medical professionals and academics^20^ can be utilised to boost research activity in primary care in the short term. Research in general practice may have the additional benefit of attracting research-interested medical students and foundation year trainees.

Further research should examine in more detail the nature of trainees’ understanding about research and patient recruitment, and determine more qualitative expressions of trainers’ perceptions of being ill-equipped to act as role models. These expanded answers (beyond this study's deduced and sometimes binary response categories) would offer policymakers more material for shaping the support required. Given that this study's findings indicated the low likelihood of patient recruitment to trials in primary care, it would be important to next understand how perceptions of research may have changed in primary care during 2020–2021, now that the need to improve evidence about the efficacy of care, services, and new drugs and vaccines is more pronounced than ever.

Primary care research can bring transformation to clinical research by presenting the opportunity for participation more widely across the region, including to many currently underserved groups within communities. Current evidence from across general practice suggests that primary care research is under-resourced, and the current model for research delivery and funding has failed to achieve its full potential. In the context of the recently published and nationally operationalised NIHR primary care research strategy,^4^ which included a theme of *Strategic Development of Primary Care Workforce*, the findings from the survey presented here has stimulated regional and national discussion about approaches to engage trainees and trainers with academic career opportunities, including academic career promotion, awareness raising of existing NIHR CRN infrastructure and identification, and showcasing research role models within the specialty. The survey results are also expected to inform regional pilots with national roll-out to enhance GP engagement through incentivisation and innovative delivery models, all with the purpose of more effectively integrating high quality research as an option of care across primary care settings.
